# Fecal Dysbiosis and Immune Dysfunction in Chinese Elderly Patients With Schizophrenia: An Observational Study

**DOI:** 10.3389/fcimb.2022.886872

**Published:** 2022-06-01

**Authors:** Zongxin Ling, Guolin Jin, Xiumei Yan, Yiwen Cheng, Li Shao, Qinghai Song, Xia Liu, Longyou Zhao

**Affiliations:** ^1^ Collaborative Innovation Center for Diagnosis and Treatment of Infectious Diseases, State Key Laboratory for Diagnosis and Treatment of Infectious Diseases, National Clinical Research Center for Infectious Diseases, The First Affiliated Hospital, School of Medicine, Zhejiang University, Hangzhou, China; ^2^ Jinan Microecological Biomedicine Shandong Laboratory, Jinan, China; ^3^ Department of Psychiatry, Lishui Second People’s Hospital, Lishui, China; ^4^ Department of Laboratory Medicine, Lishui Second People’s Hospital, Lishui, China; ^5^ Institute of Hepatology and Metabolic Diseases, Hangzhou Normal University, Hangzhou, China; ^6^ Institute of Translational Medicine, The Affiliated Hospital of Hangzhou Normal University, Hangzhou, China; ^7^ Department of Intensive Care Unit, the First Affiliated Hospital, School of Medicine, Zhejiang University, Hangzhou, China

**Keywords:** Schizophrenia, *Faecalibacterium*, LEfSe, dysbiosis, non-invasive diagnosis

## Abstract

Schizophrenia (SZ) is a severe neuropsychiatric disorder with largely unknown etiology and pathogenesis. Mounting preclinical and clinical evidence suggests that the gut microbiome is a vital player in SZ. However, the gut microbiota characteristics and its host response in elderly SZ patients are still not well understood. A total of 161 samples was collected, including 90 samples from elderly SZ patients and 71 samples from healthy controls. We explored the gut microbiota profiles targeting the V3–V4 region of the 16S rRNA gene by MiSeq sequencing, and to analyze their associations with host immune response. Our data found that bacterial β-diversity analyses could divide the SZ patients and healthy controls into two different clusters. The Linear discriminant analysis Effect Size (LEfSe) identified the compositional changes in SZ-associated bacteria, including *Faecalibacterium*, *Roseburia*, *Actinomyces*, *Butyricicoccus*, *Prevotella* and so on. In addition, the levels of pro-inflammatory cytokines such as IL-1β were greatly increased in SZ patients while the levels of anti-inflammatory cytokines such as IFN-γ were markedly decreased. Correlation analysis suggested that these bacteria contributed to immune disturbances in the host that could be used as non-invasive biomarkers to distinguish the SZ patients from healthy controls. Moreover, several predicted functional modules, including increased lipopolysaccharide biosynthesis, folate biosynthesis, lipoic acid metabolism, and decreased bile acid biosynthesis, fatty acid biosynthesis in SZ-associated microbiota, could be utilized by the bacteria to produce immunomodulatory metabolites. This study, for the first time, demonstrated the structural and functional dysbiosis of the fecal microbiota in Chinese elderly SZ patients, suggesting the potential for using gut key functional bacteria for the early, non-invasive diagnosis of SZ, personalized treatment, and the development of tailor-made probiotics designed for Chinese elderly SZ patients.

## Introduction

With the rapidly expanding global aged population, the proportion of psychiatric disorders in middle-aged and elderly individuals has dramatically increased. According to the Global Burden of Disease Study 2017 there were 1.13 million incident schizophrenia (SZ) cases in 2017 globally, while the number of newly diagnosed SZ cases was 0.29 million in China (He et al., 2020), ranking the first in the world. In 2010, there were 7.16 (6.57-7.75) million people in China affected by SZ during their lifetime (Chan et al., 2015). SZ is considered a severe and complex mental illness characterized by positive and negative symptoms, including delusions, hallucinations, thought disorder, apathy, and avolition as well as cognitive and functional impairment. Reports have shown that SZ prevalence changes with age in an inverted U-shape, with the highest value at around 40 years and nearly 71% of total cases being aged 25-54 years (Zhou et al., 2021). Due to SZ epidemiological characteristics, aging SZ patients are easy to ignore. With high stress and psychosocial burden for caregivers and a high suicide rate, SZ has become a major public problem, especially for affected elderly patients (Yu et al., 2021). Despite its low prevalence, the burden of SZ remains large and continues to increase, thereby increasing the burden on healthcare systems.

SZ is a complex psychiatric neurodevelopmental disorder with uncertain etiology. During the past decades of research, genetic, environmental, and behavioral risk factors, including development, industrialization, and urbanization have been proposed to be associated with the onset of SZ (Ma et al., 2020a). Recently, the role of non-human genetic factors such as gut microbiota has been identified in the pathophysiology of SZ (Nguyen et al., 2019; Pełka-Wysiecka et al., 2019; Li et al., 2020; Ma et al., 2020b; Pan et al., 2020; Zhu et al., 2020a; Zhu et al., 2020b; Li et al., 2021a; Yuan et al., 2021; Fan et al., 2022; Munawar et al., 2022). Well-balanced gut microbiota is critical to maintaining host general health. Mounting evidence suggests that the gut microbiota can modulate brain function and behavior *via* the “microbiota-gut-brain” axis *via* multiple routes including the vagus nerve, immune system, short-chain fatty acids and tryptophan, while its alterations may actively contribute to the SZ pathogenesis and development. Hsiao *et al.* have demonstrated that the gut microbiota can affect various complex behaviors, including social, emotional, and anxiety-like behaviors, and modulate behavioral and physiological abnormalities associated with neurodevelopmental disorders (Hsiao et al., 2013). Zhu et al. have also found that transplantation of gut microbiota from drug-free SZ patients causes SZ-like abnormal behaviors and dysregulated kynurenine metabolism in mice (Zhu et al., 2020a), which provided direct evidence that the dysbiosis of the gut microbiome may have a causal role in the development of SZ. Although most of these causal studies are conducted in animal models, they also provide valuable clues for investigating the roles and underlying mechanisms of the gut microbiota in the development of SZ. Additionally, the gut microbiota undergoes a progressive age-related physiological succession of species across the life-cycle, and the aging gut microbiota is distinct from other healthy age groups (Ling et al., 2020b). Our previous study has established the structural and functional bacterial and fungal dysbiosis of fecal microbiota in elderly patients with Alzheimer’s disease (Ling et al., 2020c; Ling et al., 2020d). Several above-mentioned studies have shown gut microbiota alterations at various stages of SZ; however, most of these participants were non-elderly SZ patients. Thus, it is important to explore the alterations of the gut microbiota in Chinese elderly SZ patients, which can elucidate the non-invasive SZ diagnosis and novel gut microbiota-based alternative therapy.

This study aimed to explore the overall structure and composition of the fecal microbiota in the elderly SZ patients without gastrointestinal diseases hospitalized in a large Chinese geriatric ward from Lishui by using the 16S rRNA gene high-throughput MiSeq platform. Additionally, we also investigated the correlations between SZ-associated key functional bacteria and clinical indicators, which would provide novel targets for the early, non-invasive diagnosis and personalized treatment of SZ and the development of tailor-made psychobiotics designed for Chinese elderly SZ patients.

## Methods

### Participants’ Enrollment

A total of 90 well-controlled Chinese elderly SZ patients (age > 62 years), who were diagnosed based on the criteria of the Diagnostic and Statistical Manual of Mental Disorders Fourth Edition (DSM-IV), were recruited from Lishui, Zhejiang province (China) from June 2020 to November 2020, with 71 age- and gender-matched cognitively normal subjects as control. These protocols for the study were approved by the Ethics Committee of Lishui Second People’s Hospital (Zhejiang, China) and written informed consent was obtained from each of the subject or their guardian before enrollment. The detailed demographic data and medical history (such as obesity, hypertension, diabetes mellitus type I or II, hypercholesterolemia, coronary heart disease, diarrhea, and constipation) were collected using a set of questionnaires. The exclusion criteria included: body mass index (BMI)  >  28 kg/m^2^; family history of dementia; any kind of other neurodegenerative disease such as Parkinson’s disease or Alzheimer’s disease; confirmed mental illness such as depression; any kind of tumor; antibiotic, prebiotic, probiotic, or synbiotic administration in the previous month; treatment with antidepressant, mood stabilizers or other psychiatric drugs in last 1 months; known active infections such as viral, bacterial, or fungal infections; other diseases such as inflammatory bowel disease, irritable bowel syndrome or other autoimmune diseases.

### Sample Collection and Bacterial DNA Extraction

Approximately 2g of a fresh fecal sample was collected in a sterile plastic cup, and stored at -80°C after preparation within 15 min until use. Serum samples from these participants were obtained using their fasting blood in the early morning. Bacterial genomic DNA was extracted from 300 mg of homogenized feces using a QIAamp^®^ DNA Stool Mini Kit (QIAGEN, Hilden, Germany) according to the manufacturer’s instructions, with additional glass-bead beating steps on a Mini-beadbeater (FastPrep; Thermo Electron Corporation, Boston, MA, USA) (Ling et al., 2020a; Ling et al., 2020d). The amount of DNA was determined using a NanoDrop ND-1000 spectrophotometer (Thermo Electron Corporation); the integrity and size were checked by 1.0% agarose gel electrophoresis containing 0.5 mg/ml ethidium bromide. All DNA was stored at -20°C before further analysis.

### Amplicon Library Construction and Sequencing

The protocols of amplicon library construction and sequencing were conducted as our previous studies (Ling et al., 2020a; Ling et al., 2020d). The details were shown as follows: amplicon libraries were constructed with Illumina sequencing-compatible and barcode-indexed bacterial PCR primers 341F (5’-CCTACGGGNGGCWGCAG-3’)/785R (5’-ACTACHVGGGTATCTAATCC-3’), which target the V3-V4 regions of the 16S rRNA gene (Fadrosh et al., 2014). All PCR reactions were performed with KAPA HiFi HotStart ReadyMix using the manufacturer’s protocol (KAPA Biosystems) and approximately 50 ng of extracted DNA per reaction. Thermocycling conditions were set at 95°C for 1 min, 55°C for 1 min, then 72°C for 1 min for 30 cycles, followed by a final extension at 72°C for 5 min. All PCR reactions were performed in 50 μl triplicates and combined after PCR. The amplicon library was prepared using a TruSeq™ DNA sample preparation kit (Illumina Inc, San Diego, CA, USA). Prior to sequencing, the PCR products were extracted with the MiniElute^®^ Gel Extraction Kit (QIAGEN) and quantified on a NanoDrop ND-1000 spectrophotometer (Thermo Electron Corporation) and Qubit 2.0 Fluorometer (Invitrogen). The purified amplicons were then pooled in equimolar concentrations and the final concentration of the library was determined by Qubit (Invitrogen). Negative DNA extraction controls (lysis buffer and kit reagents only) were amplified and sequenced as contamination controls. Sequencing was performed on a MiSeq instrument (Illumina) using a 300 × 2 V3 kit together with PhiX Control V3 (Illumina). MiSeq sequencing and library construction were performed by technical staff at Hangzhou KaiTai Bio-lab.

### Bioinformatic Analysis

The 16S rRNA gene sequence data set generated from the Illumina MiSeq platform was inputted to QIIME2 (version 2020.11), and all steps of sequence processing and quality control were performed in QIIME2 with default parameters (Caporaso et al., 2010; Ling et al., 2019; Liu et al., 2019; Ling et al., 2020a; Ling et al., 2020d). Before the following data analysis, these reads of each sample were normalized to even sampling depths and annotated using the Greengenes reference database (version 13.8) with both the RDP Classifier and UCLUST version 1.2.22 methods implemented in QIIME2. Alpha diversity, including the observed species, abundance-based coverage estimator (ACE), Chao1 estimator, Shannon, Simpson, Evenness and PD whole tree indices, was calculated at a 97% similarity level. Beta diversity was measured by the unweighted UniFrac, weighted UniFrac, jaccard and Bray-Curtis distances calculated by QIIME2, which were visualized by principal coordinate analysis (PCoA) (Lozupone and Knight, 2005). The differences in the composition of the fecal microbiota at different taxonomic levels were analyzed with Statistical Analysis of Metagenomic Profiles (STAMP) software package v2.1.3 (Parks et al., 2014) and the linear discriminant analysis (LDA) effect size (LEfSe) method (Segata et al., 2011). Krona chart was plotted using taxonomy summary data obtained from QIIME Krona chart displays abundance and hierarchy simultaneously using a radial space-filling display and features a red-green color gradient, signifying the average BLAST hits e-values within each taxon (Ondov et al., 2011). PiCRUSt v1.0.0 was used to identify predicted gene families and associated pathways from inferred metagenomes of taxa of interest identified from the compositional analyses (Langille et al., 2013). The sparse compositional correlation (SparCC) algorithm was used for correlation analysis, and the results were visualized using Cytoscape v3.4.1 (Friedman and Alm, 2012).

### Systemic Inflammatory Cytokines Analysis

Using a 27-plex magnetic bead based immunoassay kit (Bio-Rad, CA, USA), the following cytokines were quantified: interleukin-1β (IL-1β), IL-1 receptor antagonist (IL-1ra), IL-2, IL-4, IL-5, IL-6, IL-7, IL-8, IL-9, IL-10, IL-12(p70), IL-13, IL-15, IL-17, Eotaxin, Fibroblast growth factor-basic (FGF-basic), granulocyte colony-stimulating factor (G-CSF), granulocyte-macrophages colony-stimulating factor (GM-CSF), interferon gamma (IFN-γ), interferon gamma-inducible protein 10 (IP-10), monocyte chemotactic protein-1 (MCP-1), macrophages inflammatory protein-1α (MIP-1α), platelet-derived growth factor (PDGF-bb), MIP-1β, regulated upon activation normal T-cell expressed and secreted (RANTES), tumour necrosis factor-alpha (TNF-α), and vascular endothelial growth factor (VEGF). The Bio-Plex 200 system was utilized for the analysis of Bio-Rad 27-plex human group I cytokines and the Bio-Plex assay (Bio-Rad) was performed according to the manufacturer’s directions. The results expressed as picogram per milliliter (pg/mL) using standard curves integrated into the assay and Bio-Plex Manager v5.0 software with reproducible intra- and inter-assay CV values of 5-8% (Ling et al., 2020a; Ling et al., 2020c; Ling et al., 2020d).

### Statistical Analysis

White’s nonparametric *t*-test, independent *t*-test, or Mann-Whitney *U*-test were applied for continuous variables. Pearson chi-square or Fisher’s exact test were used for categorical variables between groups., Spearman’s rank correlation test was utilized for correlation analyses. Statistical analysis was performed using the SPSS v19.0 (SPSS Inc., Chicago, IL) and STAMP v2.1.3 (Parks et al., 2014). R packaged and GraphPad Prism v6.0 were used for preparation of graphs. All tests of significance were two sided, and p<0.05 or corrected p<0.05 was considered statistically significant.

### Accession Number

The sequence data from this study are deposited in the GenBank Sequence Read Archive with the accession number PRJNA807473.

## Results

### Altered Overall Structure of the Fecal Microbiota in Elderly SZ Patients

All validated SZ patients were well-controlled and not treated with psychiatric drugs in the last 1 month. Individual patient data, such as age, BMI, past medical history, and medication history were collected through the hospital medical record system. There were no significant differences in age, gender, BMI, smoking and drinking between the healthy controls and the SZ patients (p > 0.05).

**Figure 1:**
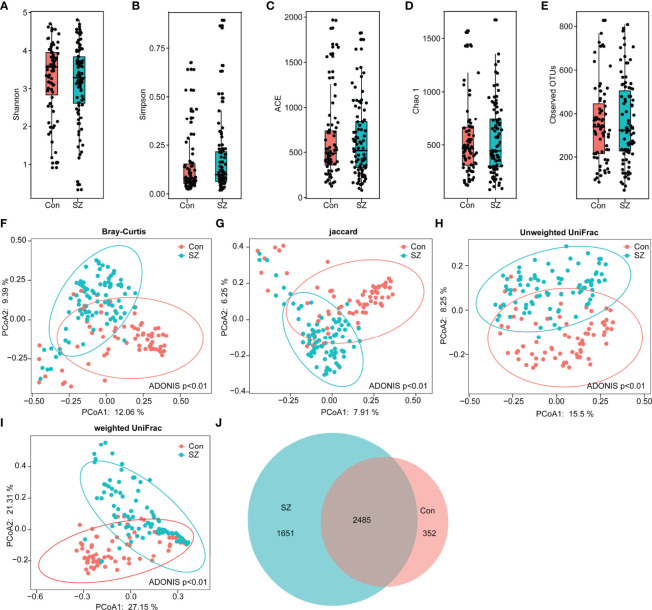
Altered overall structure of the fecal microbiota in elderly SZ patients. The diversity indices of Shannon **(A)** and Simpson **(B)**, and the richness indices of ACE **(C)**, Chao1 **(D)**, and the observed OTUs **(E)** were used to evaluate the overall structure of the fecal microbiota in the SZ patients and the healthy controls. The data are presented as mean ± standard deviation. Unpaired *t* tests (two tailed) were used to analyze the variation between the groups. Principal coordinate analysis (PCoA) plots of individual fecal microbiota based on Bray–Curtis **(F)**, Jaccard **(G)**, and unweighted **(H)** and weighted **(I)** UniFrac distances in the elderly SZ patients and the healthy controls. Each symbol represents a sample. The Venn diagram illustrates the overlap of OTUs in the SZ-associated microbiota and healthy controls **(J)**.

In our present study, we obtained 5,455,780 high-quality reads, with an average of 33,886 reads per sample, which were used for the subsequent microbiota analyses. The α-diversity indices of the SZ patients and healthy controls were analyzed based on the operational taxonomic unit (OTU) relative table. Interestingly, we identified 4,488 OTUs (unique bacterial phylotypes) in the fecal microbiota with the Good’s coverage of 98.80%, indicating that the majority of bacteria have been identified. We found that the diversity indices, such as Shannon and Simpson indices, were not significantly different between the healthy controls and the SZ patients (**Figures 1A, B**). The richness indices, such as ACE, Chao1, and observed OTUs, were also not significantly different between the SZ patients and healthy controls (**Figures 1C–E**). However, β-diversity analyses indicated that the PCoA based on the Bray-Curtis, Jaccard, unweighted UniFrac, and weighted UniFrac algorithms could divide the SZ patients and healthy controls into two different clusters despite significant interindividual variations (ADONIS test: p<0.01; **Figures 1F–I**). Additionally, a Venn diagram showed that 2,485 of 4,488 OTUs were detected in the two groups, while 1,651 and 352 OTUs were unique to patients with SZ and healthy controls, respectively (**Figure 1J**). Our data suggested that the overall structure of the SZ-associated fecal microbiota was obviously altered compared with that of the healthy controls.

### Altered Fecal Microbiota Profiles in the Elderly SZ Patients

**Figure 2:**
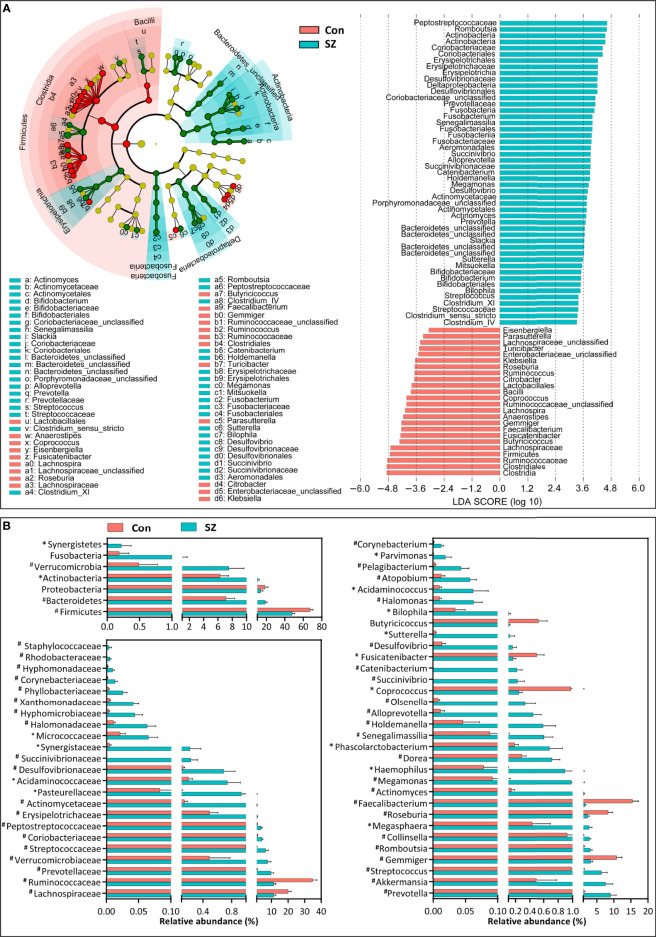
Differential bacterial taxa between the elderly SZ patients and the healthy controls. The LEfSe identified the taxa with the greatest differences in abundance between the elderly SZ patients and the healthy controls. Only the taxa meeting a significant LDA threshold value of > 3 are shown **(A)**. Comparisons of the relative abundance of the abundant bacterial taxa at the level of bacterial phylum, family, and genus **(B)**. The data are presented as the mean ± standard deviation. Mann–Whitney *U*-tests were used to analyze variation between the elderly SZ patients and the healthy controls. *p < 0.05 and ^#^p < 0.01 compared with the control group.

**Figure 3:**
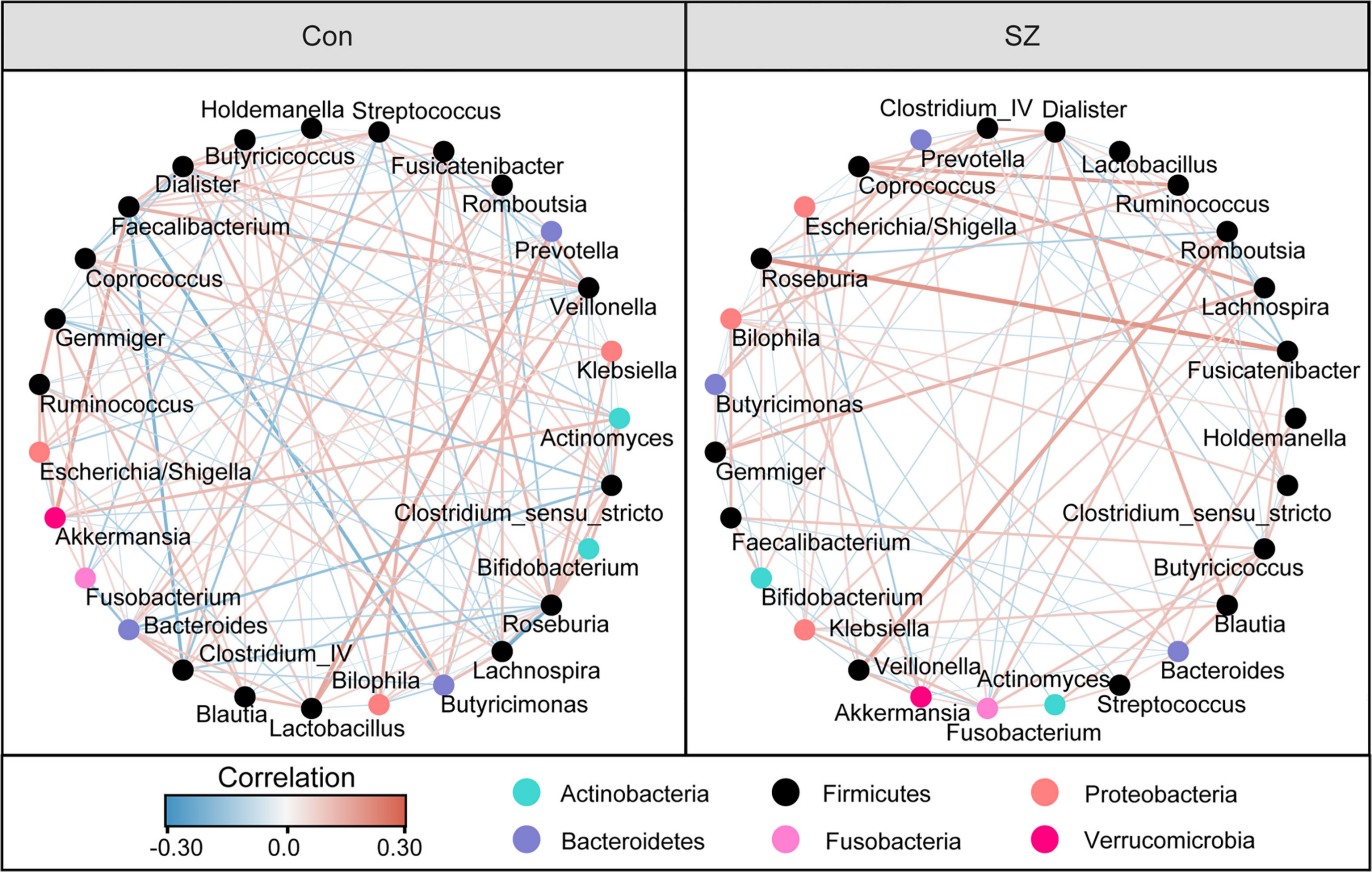
Strengths of the correlation between abundant fecal microbiota in the elderly SZ patients and the healthy controls. Correlation network of the abundant fecal microbiota in the healthy controls and the elderly SZ patients. The correlation coefficients were calculated with the Sparse Correlations for Compositional (SparCC) data algorithm. Cytoscape version 3.4.0 was used for network construction. The red and blue lines represent positive and negative correlations, respectively. The correlation network became simpler in the elderly SZ patients.

Differential abundance testing at different taxonomic levels was performed to identify the drivers of community separation. The compositions of the fecal microbiota in the SZ patients and the healthy controls from phylum to genus level are demonstrated in **Supplementary Figure 1**. Using the RDP classifier, the sequencing reads were classified as 12 phyla, 78 families, and 253 genera. In addition, Krona radial space-filling charts showed the mean relative abundances of bacterial taxa in healthy controls and SZ patients from phylum to genus level (starting at the inner circle) (**Supplementary Figure 2**). By using the LEfSe, our discriminant analyses showed that many key functional taxa were obviously different between the SZ patients and the healthy controls (LDA score > 3, p < 0.05, **Figure 2A**). Only bacterial phylotypes with an average relative abundance of more than 0.01% were selected for the LEfSe. The representative cladogram demonstrated the dysbiosis of SZ-associated fecal microbiota in the Chinese SZ patients. Specifically, the relative abundance of microbiota at the phylum level showed that the proportion of Firmicutes significantly decreased in SZ patients while that of Bacteroidetes, Actinobacteria, Verrucomicrobia and Synergistetes increased (p < 0.05, **Figure 2B**). At the family level, the proportion of Lachnospiraceae and Ruminococcaceae was decreased significantly while that of others families, such as Prevotellaceae, Verrucomicrobiaceae, and Streptococcaceae, increased in SZ patients (p < 0.05, **Figure 2B**). As far as the genus level was concerned, the proportion of six genera, such as *Gemmiger*, *Roseburia*, *Faecalibacterium*, *Coprococcus*, *Fusicatenibacter* and *Butyricicoccus*, significantly reduced, while that of the other 26 functional genera, including *Prevotella*, *Akkermansia*, *Streptococcus*, *Romboutsia* and so on dramatically increased in SZ patients (p < 0.05, **Figure 2B**). These data indicated that differences in the overall composition of the fecal microbiota between SZ patients and healthy controls were evident. Moreover, the structure of the fecal microbiota in both groups was determined by dynamic interactions between these altered functional genera. In addition, the structure of the fecal microbiota was determined by dynamic interactions between these community members. Our SparCC algorithm with FDR adjustments was used to generate correlation-based networks of microbial interaction based on the relative abundance of OTUs between the groups (**Figure 3**). A more complex network of interactions in healthy controls were found than that in the SZ patients. We also identified more positive and negative correlations among the bacteria in the healthy controls compared to that in the SZ patients. These results indicated microbial dysbiosis in SZ-associated fecal microbiota.

### Fecal Microbiota-Based Signature Discriminated SZ From Controls

**Figure 4:**
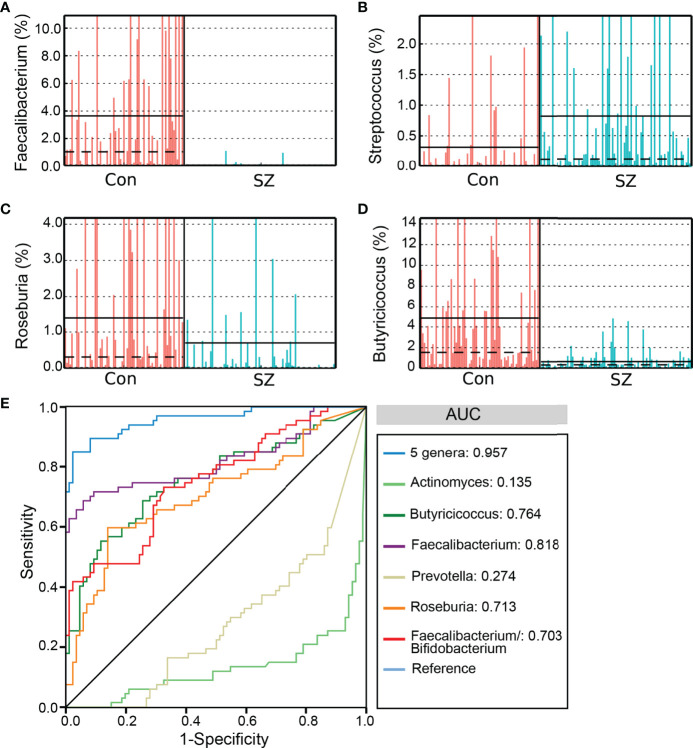
The differential genera as SZ diagnostic markers. The relative abundance of the differential genera such as *Faecalibacterium* **(A)**, *Streptococcus* **(B)**, *Roseburia* **(C)**, and *Butyricicoccus* **(D)** in each sample. Receiver operating characteristic (ROC) curves for the differential genera alone or in combination, and *Faecalibacterium/Bifidobacterium*, used to discriminate SZ patients from healthy controls **(E)**. AUC, the area under the receiver operating characteristic curve.

As mentioned above, the SZ-associated fecal microbiota showed significant microbial dysbiosis. Based on the LEfSe, several genera could be used as potential biomarkers to discriminate SZ patients from healthy controls. We assessed the potential discriminating value using several differential genera as biomarkers, such as *Faecalibacterium*, *Roseburia*, *Actinomyces*, *Butyricicoccus*, and *Prevotella*, and the *Faecalibacterium/Bifidobacterium* ratio. The differential features of the predominant genera, including *Faecalibacterium*, *Streptococcus*, *Roseburia*, *Butyricicoccus*, are shown in **Figures 4A–D**, which demonstrates significant interpersonal variations. With only one of the differential bacteria as a predictor, we generated the area under the receiving operating characteristic curves to obtain the area under the curve (AUC) ranging between 0.135-0.818 (**Figure 4E**). Our study indicated that decreased *Faecalibacterium* in SZ patients was the best discriminant predictor for the healthy controls (AUC: 0.818), while increased *Actinomyces* in SZ patients was the best discriminant predictor for SZ patients (AUC: 0.865). Furthermore, a multivariable stepwise logistic regression analysis was applied to the list of SZ-associated genera to determine the taxa that best distinguished the healthy controls from the SZ patients. We found that using all five aforementioned abundant genera significantly improved predictive performance (AUC: 0.957). Thus, these key functional differential genera could distinguish between the SZ and healthy control groups among our cohort, indicating that the fecal microbiota could be used as potential biomarkers to predict SZ.

### Microbial Functional Dysbiosis in SZ

**Figure 5:**
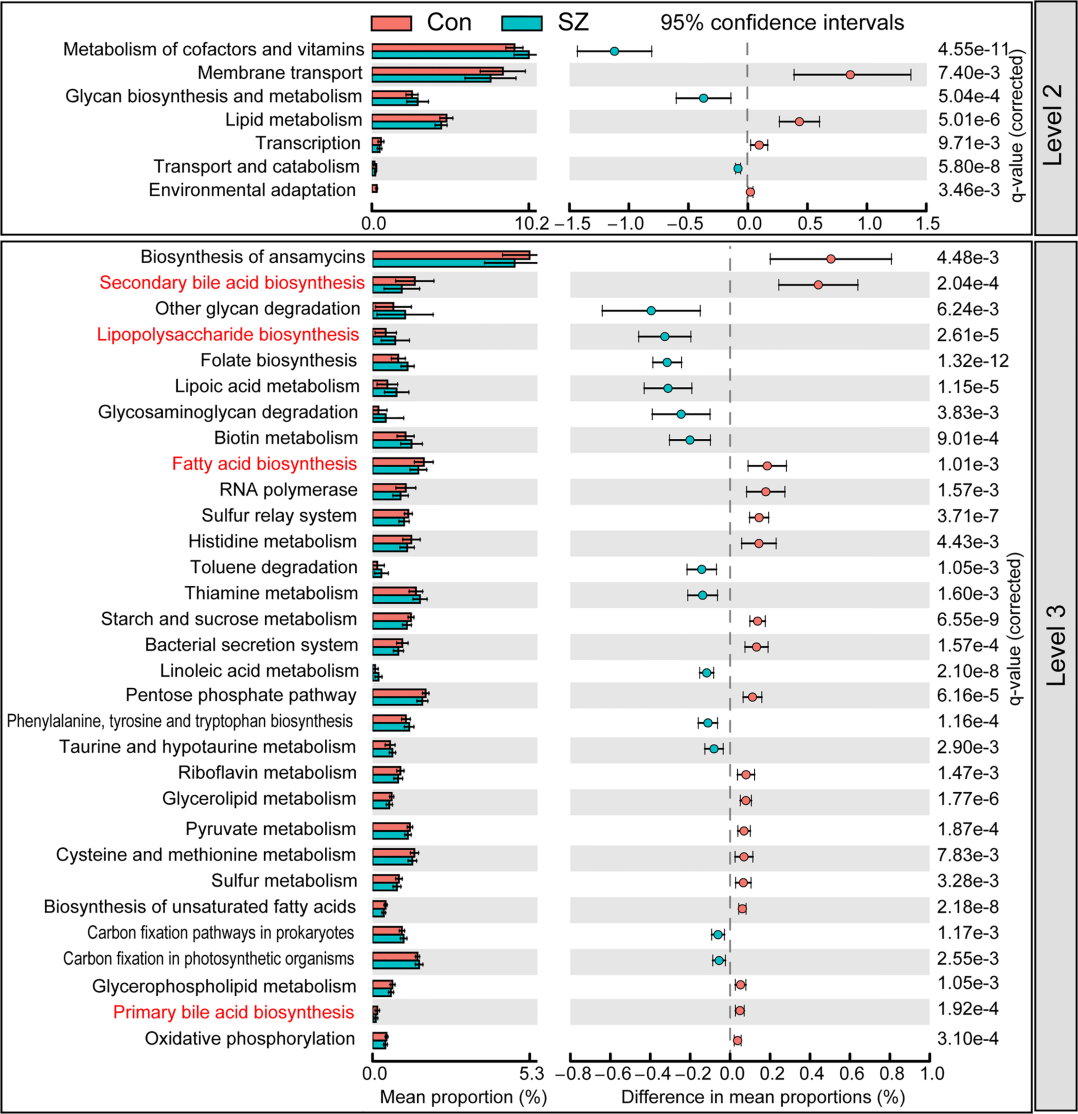
PiCRUSt-based examination of the fecal microbiome of the elderly SZ patients and the healthy controls. The different bacterial functions were evaluated between them based on two-sided Welch’s *t*-test. Comparisons between the groups for each KEGG functional category (levels 2 and 3) are shown by percentage. The Benjamini–Hochberg method was used for multiple testing correction based on the false discovery rate (FDR) through STAMP.

PiCRUSt was used to predict the abundances of functional categories the Kyoto Encyclopedia of Genes and Genomes (KEGG) ortholog (KO) based on closed-reference OTUs picking to identify the metabolic and functional changes in the fecal microbiota between the SZ patients and the healthy controls. We compared 64 KEGG pathways at level 2 and identified seven categories with clearly differential abundances between the SZ patients and the controls. Metabolism of cofactors and vitamins, glycan biosynthesis and metabolism, transport and catabolism significantly increased in SZ patients, while membrane transport, lipid metabolism, transcription and environmental adaptation significantly decreased (p < 0.05; **Figure 5**). A total of 31 pathways in level 3 were identified with significantly different abundances in the fecal microbiome between the SZ patients and healthy controls (p < 0.05). Specifically, 13 pathways including lipopolysaccharide biosynthesis, folate biosynthesis, and lipoic acid metabolism, showed higher activity in SZ-associated fecal microbiota, while 18 other pathways, including secondary bile acid biosynthesis, fatty acid biosynthesis, and primary bile acid biosynthesis, showed a prominently decreased activity in the SZ-associated fecal microbiota. Collectively, these data indicate that the functional dysbiosis of the SZ-associated fecal microbiota may participate in the pathogenesis and development of SZ.

### Correlations Between Differential Genera and Host Cytokines

**Figure 6:**
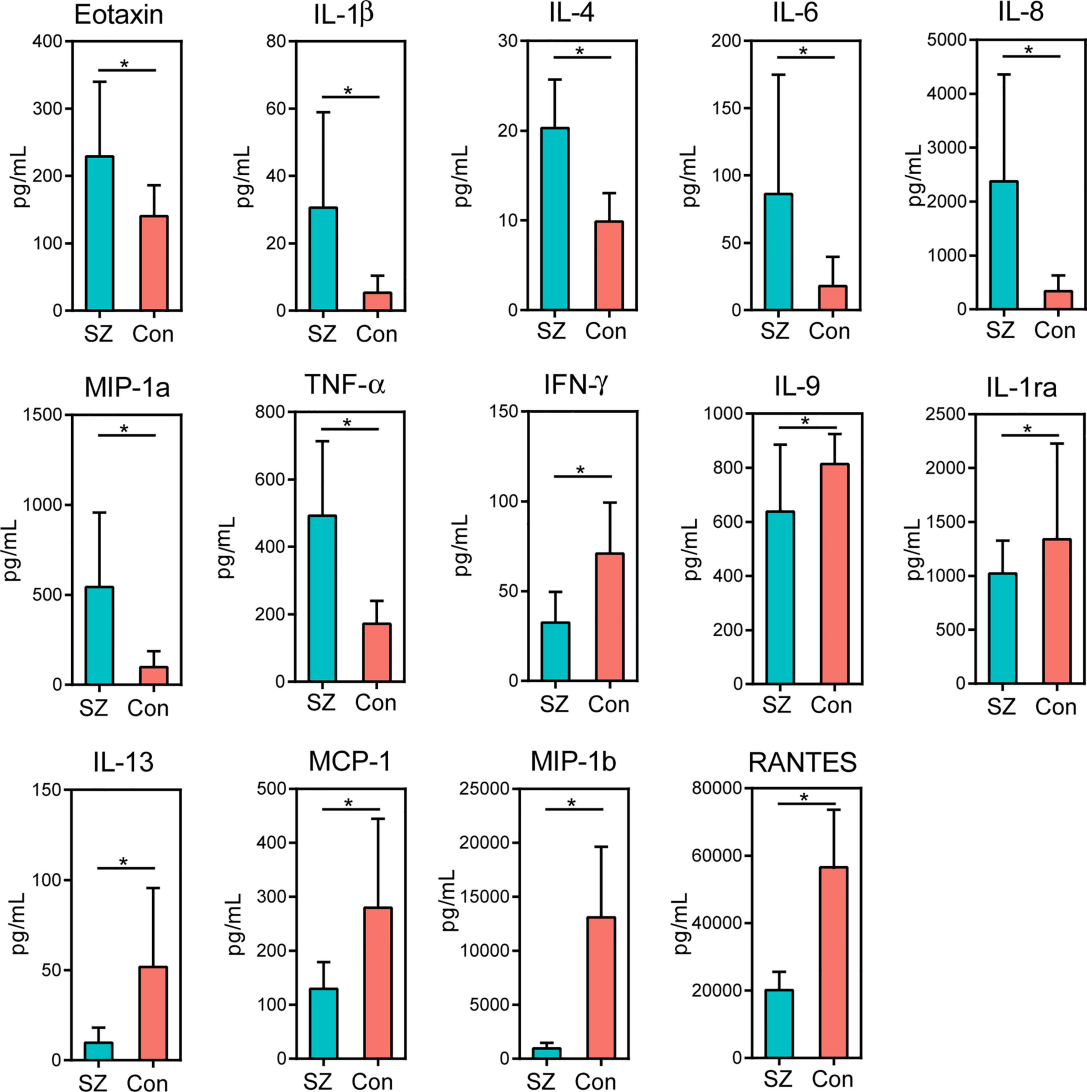
Mean (SEM) concentrations (pg/ml) of 27 pro- and anti-inflammatory cytokines and chemokines in the elderly SZ patients and in healthy controls determined using Bio-Plex immunoassays. The concentrations of Eotaxin, IL-1β, IL-4, IL-6, IL-8, MIP-1a and TNF-α increased significantly in the elderly SZ patients, while those of IFN-γ, IL-9, IL-1ra, IL-13, MCP-1, MIP-1b and RANTES decreased significantly. *p < 0.05.

**Figure 7:**
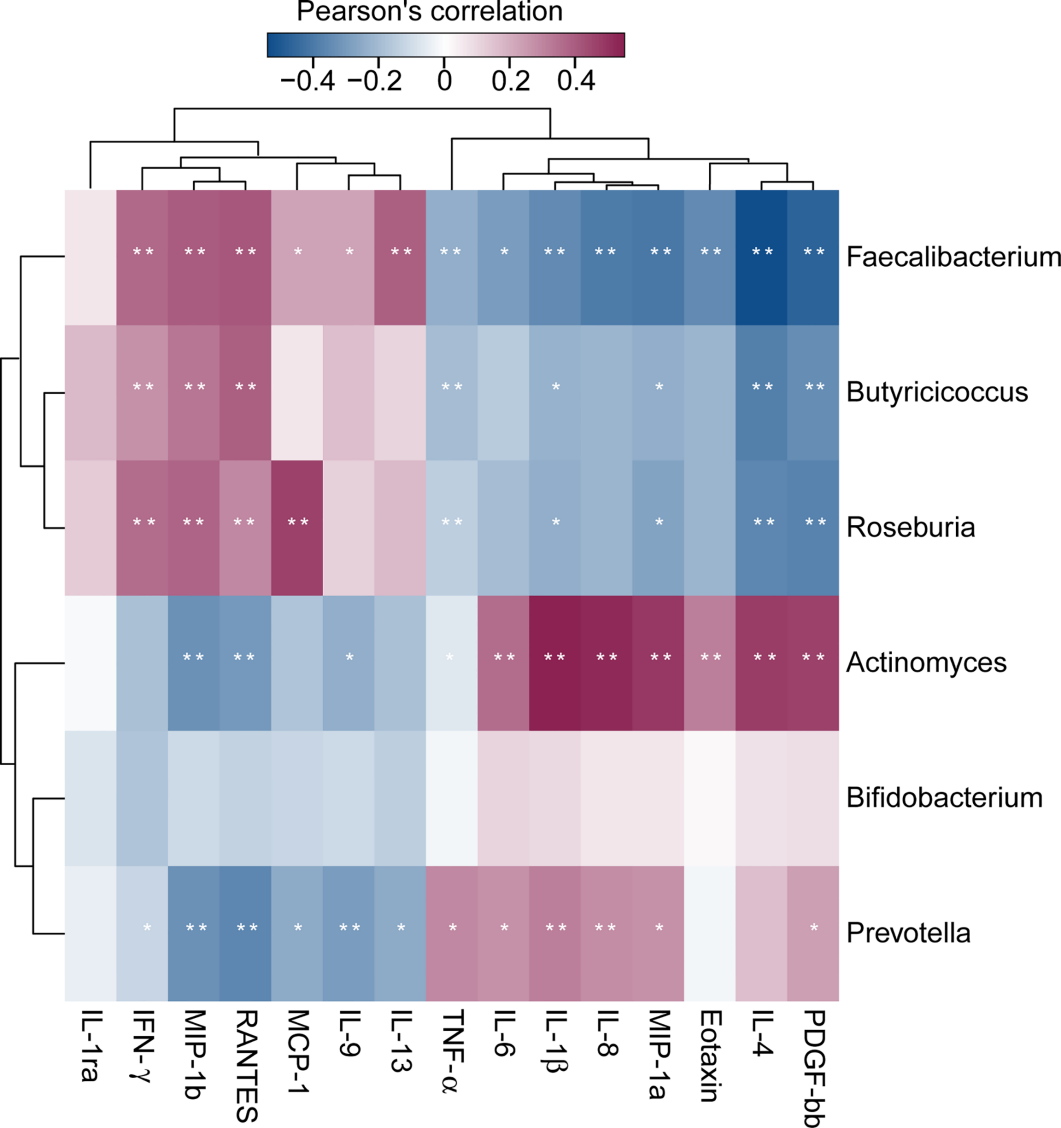
Correlation between fecal microbiota, and pro- and anti-inflammatory cytokines and chemokines and clinical indicators. The heatmap shows partial Spearman’s correlation coefficients between 6 genera and host immunity in SZ patients. Spearman’s rank correlation (r) and probability (p) were used to evaluate statistical importance. *p < 0.05; **p < 0.01.

Of the 27 cytokine levels examined in the present study, seven cytokines, including eotaxin, IL-1β, IL-4, IL-6, IL-8, MIP-1a and TNF-α, were greatly increased in SZ patients relative to healthy controls, while other seven cytokines, such as IFN-γ, IL-9, IL-1ra, IL-13, MCP-1, MIP-1b and RANTES, were markedly decreased in SZ patients (**Figure 6**, p < 0.05 for each). These alterations in pro- and anti-inflammatory cytokines and chemokines indicated complex immune dysfunction in these SZ patients. We performed a correlation analysis using Pearson’s correlation to determine the associations between the deferential genera of the SZ patients and the altered cytokines (**Figure 7**). We found that those butyrate-producing genera, such as *Faecalibacterium*, *Roseburia*, and *Butyricicoccus*, were negatively associated with the above-mentioned increased cytokines in SZ patients and positively correlated with the above-mentioned decreased cytokines. Different from those aforementioned correlations, the genera that were increased in SZ patients, such as *Actinomyces* and *Prevotella*, showed converse correlations with these altered cytokines and chemokines. The altered fecal microbial profiles and their related host immunity might actively participate in the pathophysiology of SZ.

## Discussion

During the past decade, the new concept “microbiota-gut-brain axis” strongly connected the gut microbiota and the brain function emerging as a new focal point of biomedical research. A growing body of evidence indicates that the gut microbiota plays a crucial role in the complex bidirectional communication between the gut and the brain, which profoundly influences neural development, neuroinflammation, activation of the stress response, and neurotransmission, in addition to modulating complex behavior, such as sociability and anxiety. Since 2006, multi-omics sequencing and analytical technologies have provided valuable insights regarding the taxonomic diversity of the gut microbiota on a much deeper level and demonstrated its novel associations with human diseases. Recent advances in gut microbiota studies have expanded beyond simply profiling microbiota compositions and have been increasingly characterizing microbial functions by using functional meta-omics approaches and deciphering the cross-talk between the gut microbiota and host in detail Ling et al., 2022. With these developed omics techniques, our previous studies have found that the gut microbiota dysbiosis leading to changes in the bidirectional relationship between gut microbiota and the nervous system is linked to the pathogenesis of several neuropsychiatric diseases, such as major depressive disorder, Alzheimer’s disease, Parkinson’s disease, multiple sclerosis and so on (Jiang et al., 2015; Ling et al., 2020a; Ling et al., 2020c; Ling et al., 2020d; Sun et al., 2020; Sun et al., 2021; Wu et al., 2021). Different from previous genetic and environmental findings, the alterations of the gut microbiota provide novel insights into the pathogenesis of these neuropsychiatric disorders. The dysfunctional gut microbiota can translate into brain phenotypes including psychiatric symptoms and cognitive deficits via the microbiota-gut-brain axis. A previous study has shown that the gut microbiota can control the expression of various neurotrophic factors, such as brain-derived neurotrophic factor (BDNF), which can affect neural development and brain plasticity (Sudo et al., 2004). Recently, Li *et al.* have also observed a correlation between the gut microbiota and the brain structure and function in SZ (Li et al., 2021a). Since the gut microbiota is an ecosystem adjustable by genetic and environmental factors, these altered gut key functional bacteria can be used as novel targets for the diagnosis and treatment of psychiatric disorders and clarify their pathogeneses.

Being different from most previous microbiome studies on young and adult SZ patients, this study enrolled elderly SZ patients to explore the alterations of the gut microbiota and the host immune response. During the aging process, human gut microbiota underwent physiological succession and changed gradually across the life cycle. Compared with the age-matched elderly healthy controls, we found that the overall structure of the fecal microbiota (β-diversity) was different between SZ patients and healthy controls, although the α-diversity indices were not significantly different between the two groups. However, Yuan et al. have demonstrated that the first-episode unmedicated young SZ patients showed lower α-diversity (the Shannon and Simpson’s indices) compared to healthy controls (Yuan et al., 2021). The disparity between the two Chinese studies might have been caused by age-related differences in the gut microbiota baseline. Similar to our findings, Nguyen et al. have also found that the α-diversity indices were not significantly changed in the adult SZ Americans (Nguyen et al., 2021). Antipsychotics can influence the gastrointestinal hypomotility of SZ patients, leading to antipsychotic-induced constipation. Xu et al. have found that the antipsychotic-induced constipation group had a significantly increased α-diversity in observed species, Chao 1, and ACE as compared to the non-constipation group (Xu et al., 2021). The altered microbiota diversity might affect the gut-brain axis and influence the etiology and clinical manifestation of SZ.

Additionally, our results from 16S rRNA sequencing demonstrated significant changes in microbial composition in elderly SZ patients. SZ was associated with profound changes in the fecal microbiota, with an increase in Bacteroidetes and Actinobacteria and a decrease in Firmicutes on the phylum level. Although the changing patterns were not always similar to our findings, other previous studies have also shown that the compositional changes of the human gut microbiota were closely linked to SZ (Schwarz et al., 2018; Shen et al., 2018; Zheng et al., 2019; Zhu et al., 2020b). Indeed, the relative abundances and differences in taxa have shown extensive variation from study to study. In elderly SZ patients, we found that the abundance of several representative butyrate-producing genera, such as *Roseburia*, *Faecalibacterium*, *Coprococcus* and *Butyricicoccus*, were significantly reduced. Interestingly, our previous microbiome studies in multiple sclerosis and AD have also demonstrated similar changing microbiota profiles (Ling et al., 2020a; Ling et al., 2020d). Our correlation analyses also found that these butyrate-producing bacteria were positively and negatively correlated with the pro- and anti-inflammatory cytokines, respectively, indicating that these bacteria can modulate host immune response. Our findings suggested that these representative butyrate-producing genera might play crucial roles in preventing these neuropsychiatric diseases, although the biological role of these key functional bacteria still needs to be clarified deeply. In fact, the PiCRUSt analysis also revealed the altered functional pathways, such as fatty acid biosynthesis, in the SZ-associated microbiota. Short-chain fatty acids (SCFAs), primarily acetate, propionate, and butyrate, were produced by various commensal bacteria in the human gut. SCFAs production and regulation have been proposed as one of the mechanisms by which probiotics promote health outcomes (Gargari et al., 2016). The imbalance of the gut microbiota in elderly SZ patients, especially the reduction of butyrate-producing bacteria, was accompanied by a decrease in the synthesis of butyrate subsequently. Among these three major SCFAs, butyrate has received particular attention for its beneficial effects on gut homeostasis and energy metabolism. Numerous *in vitro* and *in vivo* studies have shown that butyrate plays a vital role in modulating immune and inflammatory responses and gut mucosal barrier function (Tan et al., 2014). Recently, butyrate has been demonstrated to act as a signaling molecule to communicate between the gut, gut microbiota, and the brain, which can directly or indirectly modulate brain functions through immune, endocrine, vagal, and other humoral pathways. There is ample evidence showing that butyrate can cross the blood-brain barrier, and its concentrations in wet brain samples are about an order of magnitude higher than those in the peripheral blood, which will help to shape the brain immune milieu (Kim et al., 2013; Sun et al., 2016). Greenhill et al. have found that butyrate plays an essential role in regulating synaptic plasticity and cortical development (Greenhill et al., 2015). Animal studies have also shown that modulation of butyrate can improve cognitive performance and influence hippocampal neurogenesis (Liu et al., 2015; Val-Laillet et al., 2018; Yang et al., 2018). The impact of gut-derived butyrate on the brain has reinforced the notion of the existence of the microbiota-gut-brain axis. The level of butyrate in the gut and brain may be critical in determining the effects on behavioral and psychophysiological processes. Stilling et al. have found that supraphysiological doses of butyrate exert potent neuropharmacological effects, facilitating synaptic tagging and capturing (Stilling et al., 2016). A previous study has demonstrated that butyrate can attenuate microglia-mediated neuroinflammation in Alzheimer’s disease by regulating the microbiota-gut-brain axis (Sun et al., 2020). Li et al. have observed that increased serum levels of butyrate after 24-week risperidone treatment might be associated with a favorable treatment response (the reduction in the PANSS score) in drug-naïve, first-episode SZ (Li et al., 2021b). With risperidone monotherapy, the increased butyrate in the serum might be associated with the increased relative abundance of butyrate-producing bacteria in the gut in SZ patients. Our previous study has identified that administration of *Clostridium butyricum* can increase butyrate levels in feces and in the brain in a mouse model of vascular dementia, resulting in significant attenuation of cognitive impairment and histopathological changes in the hippocampus (Liu et al., 2015). An open-label inpatient pilot clinical trial has found that oligofructose-enriched inulin can increase serum butyrate concentrations in SZ patients and modulate cognitive function in visual learning, processing speed, and attention positively (Kelly et al., 2021). The butyrogenic prebiotic fibers can promote the increase in gut-derived butyrate by increasing the relative abundance of butyrate-producing bacteria. In addition, diet, especially the healthiest Mediterranean diet, in which a person consumes mainly plant-based food, can increase the butyrate-producing bacteria in the gut, thereby modulating butyrate production (Nagpal et al., 2019). These studies have suggested that butyrate supplementation directly or increased butyrate-producing bacteria *via* manipulating the gut microbiota indirectly can be utilized as a promising and convenient approach to treat SZ by targeting microbiota-gut-brain interactions in the elderly.

Other differential functional bacteria, such as *Prevotella*, *Akkermansia*, *Streptococcus*, *Romboutsia* and so on, were increased in elderly SZ patients. These genera, such as *Prevotella* and *Actinomyces*, were negatively and positively correlated with anti-inflammatory and pro-inflammatory cytokines, respectively, suggesting that these genera might actively participate in the pathogenesis of SZ. These correlation analyses are almost opposite to the findings in butyrate-producing bacteria. These significant increments in SZ genera might promote neuroinflammation and then contribute to cognitive dysfunction, a decline in motivation, and psychosis. *Prevotella*, a genus with high genetic diversity within and between species, has been considered commensal bacteria due to its extensive presence in the healthy microbiota. Our previous study has also found a decreased proportion of *Prevotella* in patients with multiple sclerosis (Ling et al., 2020a), whereas its abundance increased after successful treatment. However, increasing evidence has indicated that *Prevotella* (at least some strains) has been considered clinically important pathobionts that can participate in human disease by promoting chronic inflammation (Ley, 2016). *Prevotella* exhibits increased inflammatory properties, as demonstrated by the augmented release of inflammatory mediators from immune cells and various stromal cells (Larsen, 2017). There has been compelling mechanistic and causal evidence in mice that *Prevotella* can promote inflammatory disease features. By activating toll-like receptor 2, *Prevotella* can lead to the production of Th17-polarizing cytokines by antigen-presenting cells, including IL-23 and IL-1. Recently, Agarwala et al. have also shown a five-fold increase in *Prevotella* in autistic children (Agarwala et al., 2021). *Prevotella*-mediated local inflammation leads to systemic dissemination of inflammatory mediators, bacteria, and bacterial products, which in turn may affect gut-brain disorder outcomes. There is still a need for more studies to ascertain a causal and potential SZ-triggering role of *Prevotella*, which can help to open up novel therapeutic targets for SZ. Another SZ-associated genus, intestinal mucin layer-degrading *Akkermansia*, was significantly increased in elderly SZ patients. Similar to our previous study, we have also found increased *Akkermansia* in other gut-brain disorders, such as AD (Ling et al., 2020d). *Akkermansia*, an acetate producer, has been considered a next-generation probiotic candidate with significant prospects for its application. The beneficial roles of *Akkermansia* (typical strain is *Akkermansia muciniphila*) in metabolic diseases, inflammatory bowel disease and tumor immunotherapy has been widely recognized. However, this study found that *Akkermansia* should not always be considered a potentially beneficial bacterium since it might be harmful for the gut-brain axis in the context of SZ development in the elderly. Our previous study has also found that the traditional probiotic *Bifidobacteria*, another acetate producer, also participated in the development of AD (Ling et al., 2020d). Nishiwaki et al. have demonstrated that *Akkermansia* is significantly increased in patients with PD across countries (Nishiwaki et al., 2020). *Akkermansia* can degrade the mucus layer of the gut, leading to increased intestinal permeability (gut leakiness) in these elderly patients (Forsyth et al., 2011).

Previous studies have shown that increased bacterial translocation due to the increased intestinal permeability and innate immune imbalance are both presented in patients with SZ (Severance et al., 2013; Maes et al., 2019). As aforementioned, the different SCFAs profiles produced by these key functional bacteria might play distinct roles in the pathophysiology of various neurological conditions. Specifically, butyrate, but not acetate, can improve gut integrity, attenuate pro-inflammatory cytokine expression in microglia, and reduce peripheral inflammation concurrent to decreased brain neuroinflammation (Matt et al., 2018). A previous study has identified that increased acetate mediates the microbiome-brain-β-cell axis to promote metabolic syndrome (Perry et al., 2016). Pérez-Pérez et al. have also found that plasma acetate correlates with disability and immune response in multiple sclerosis (Pérez-Pérez et al., 2020). In combination with our previous microbiome studies in gut-brain disorders, the discrepancy in SCFAs profiles provided clues to how the gut microbiota might modulate the pathophysiology of SZ. Further studies are warranted to elucidate the relationships between gut-derived SCFAs and neuroinflammation in these elderly SZ patients. These new findings will elucidate the novel therapeutic options for these elderly SZ patients targeting the regulation of the SCFAs profiles.

The current diagnostic criteria of SZ emphasize mainly clinical symptoms and signs. However, the absence of pathognomonic clinical features or specific laboratory tests has made SZ diagnosis more subjective. Thus, it is urgent to find biomarkers for SZ diagnosis. This study found that bacterial dysbiosis was involved in the development of SZ in the elderly. The characteristics of the gut microbiota could be used as a clinical biomarker for SZ. These differential key functional genera, such as *Faecalibacterium*, *Roseburia*, *Actinomyces*, *Butyricicoccus*, and *Prevotella*, and the *Faecalibacterium/Bifidobacterium* ratio could be used as biomarkers to distinguish between SZ and healthy controls. Interestingly, the five genera in combination could significantly improve predictive performance when compared with those single genera. In another SZ metagenomic study, Zhu et al. have constructed and validated a discriminatory model using the deferential microbial species, which can clearly distinguish SZ patients from healthy controls (Zhu et al., 2020b). This discriminatory model provides an accurate and objective method for the diagnosis of SZ. Our previous studies have also shown that those deferential key functional genera could be used as diagnostic biomarkers for the diagnosis of neuropsychiatric diseases (Ling et al., 2020a; Ling et al., 2020d). In combination with other indicators, the gut microbiota, especially these key functional bacteria, could help to improve the accuracy of SZ diagnosis.

However, several limitations of this study should be mentioned. First, analysis of the bacterial 16S rRNA gene V3-V4 regions had a limited resolution in terms of bacterial species identification. The 16S rRNA gene amplicon sequencing used in this study generally captured reliable taxonomic classification at the genus level. Further metagenomic studies in elderly SZ patients would provide more information on the alterations of the gut microbiota. Second, this was a single-center study with a small sample size. Future multicenter microbiota studies should include larger cohorts to verify our conclusions. Third, this study only identified several SZ-associated key functional bacteria and predicted their roles in the development of SZ. However, their biological role requires further investigation in future *in vitro* and *in vivo* studies. Furthermore, clinical studies with fecal microbiota transplantation or specific psychobiotic strains administration might help to clarify the relationship between the gut microbiota and SZ more clearly.

In summary, the gut microbiota in elderly SZ patients significantly changed when compared with healthy controls. Although the α-diversity indices did not alter significantly, the β-diversity analysis, such as PCoA, could divide the SZ patients and healthy controls into two different clusters, suggesting structural dysbiosis in SZ patients. The LEfSe identified several changing genera, such as *Faecalibacterium*, *Roseburia*, *Actinomyces*, *Butyricicoccus*, *Prevotella*, and so on, demonstrating that the differences in the overall composition of the fecal microbiota between SZ patients and healthy controls were evident. These altered key functional bacteria could be used as non-invasive biomarkers to discriminate SZ patients from healthy controls. Targeting on these altered key functional bacteria through a personalized diet or intervention from beneficial microbiota may be useful for patient-tailored early intervention in SZ cases. In addition, the functional dysbiosis in SZ-associated fecal microbiota also suggests that the changed fecal microbiota is associated with decreased fatty acid biosynthesis and bile acid biosynthesis, and is correlated with the altered host immunity, which might play vital roles in the pathogenesis and development of SZ. Thus, our investigation of the fecal microbiota using a large and confirmed SZ cohort provides novel insights into disease pathogenesis, which could provide new approaches to managing neurodevelopmental disorders by modulating the gut microbiome.

## Data Availability Statement

The datasets presented in this study can be found in online repositories. The names of the repository/repositories and accession number(s) can be found in the article/**Supplementary Material**.

## Ethics Statement

The studies involving human participants were reviewed and approved by the Ethics Committee of Lishui Second People’s Hospital (Zhejiang, China). The patients/participants provided their written informed consent to participate in this study.

## Author Contributions

ZL, XL and LZ conceived and designed the experiments. ZL, GJ, XY, YC, LS, XL and QS performed the experiments. ZL, LS and XL analyzed the data. ZL, LS and XL wrote the paper and edited the manuscript. The final manuscript was read and approved by all authors. All authors contributed to the article and approved the submitted version.

## Funding

This present work was funded by the grants of Key R&D Program of Zhejiang (2022C03060), the Research Fund for Lin He Academician New Medicine (17332005), the S&T Major Project of Lishui (2017ZDYF15), the National Natural Science Foundation of China (81771724, 31700800, 81790631), Zhejiang Basic Public Welfare Research Project (LGF20H090016), the Research Project of Jinan Microecological Biomedicine Shandong Laboratory (JNL-2022033C), the Taishan Scholar Foundation of Shandong Province (tsqn202103119), the Nutrition and Care of Maternal & Child Research Fund Project of Guangzhou Biostime Institute of Nutrition & Care (2019BINCMCF045), the National S&T Major Project of China (2018YFC2000500), and the Foundation of China’s State Key Laboratory for Diagnosis and Treatment of Infectious Diseases.

## Conflict of Interest

The authors declare that the research was conducted in the absence of any commercial or financial relationships that could be construed as a potential conflict of interest.

## Publisher’s Note

All claims expressed in this article are solely those of the authors and do not necessarily represent those of their affiliated organizations, or those of the publisher, the editors and the reviewers. Any product that may be evaluated in this article, or claim that may be made by its manufacturer, is not guaranteed or endorsed by the publisher.

## Supplementary Material

Supplementary Figure 1 Variations in the compositions of fecal microbiota in the elderly SZ patients and the healthy controls in the phylum, class, order, family, and genus levels.

Supplementary Figure 2 Krona charts showing the fecal microbiota in elderly SZ patients and healthy controls. These taxa represent the internal core microbiota at the individual level.
