# Supplementary figures and images for: Fecal Dysbiosis and Immune Dysfunction in Chinese Elderly Patients With Schizophrenia: An Observational Study
Source: Front Cell Infect Microbiol. 2022 Jun 1;12:886872. doi: 10.3389/fcimb.2022.886872 (PMC9198589; doi:10.3389/fcimb.2022.886872)

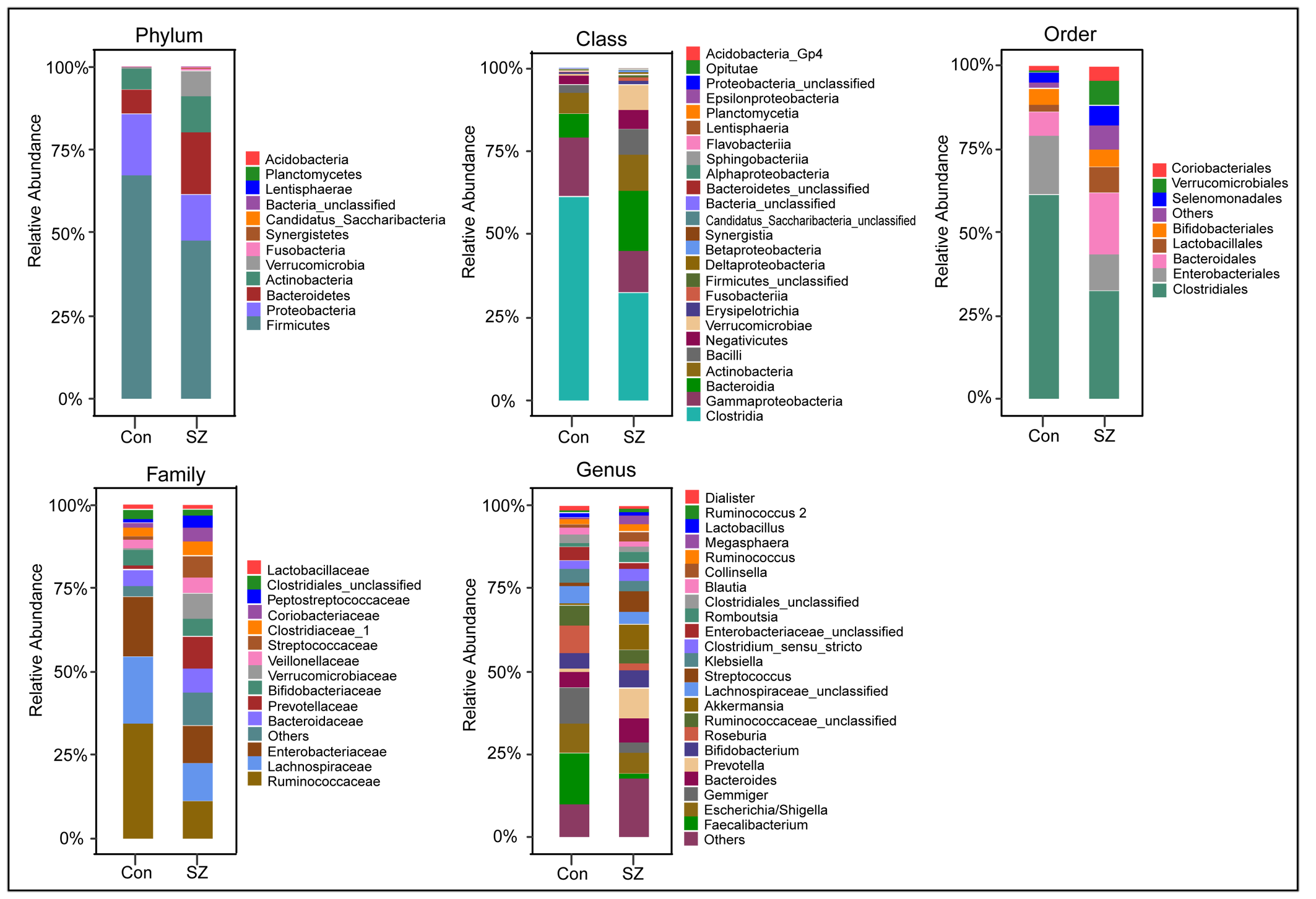

Supplement: Supplementary Figure 1 — Variations in the compositions of fecal microbiota in the elderly SZ patients and the healthy controls in the phylum, class, order, family, and genus levels. [file Image_1.tif]

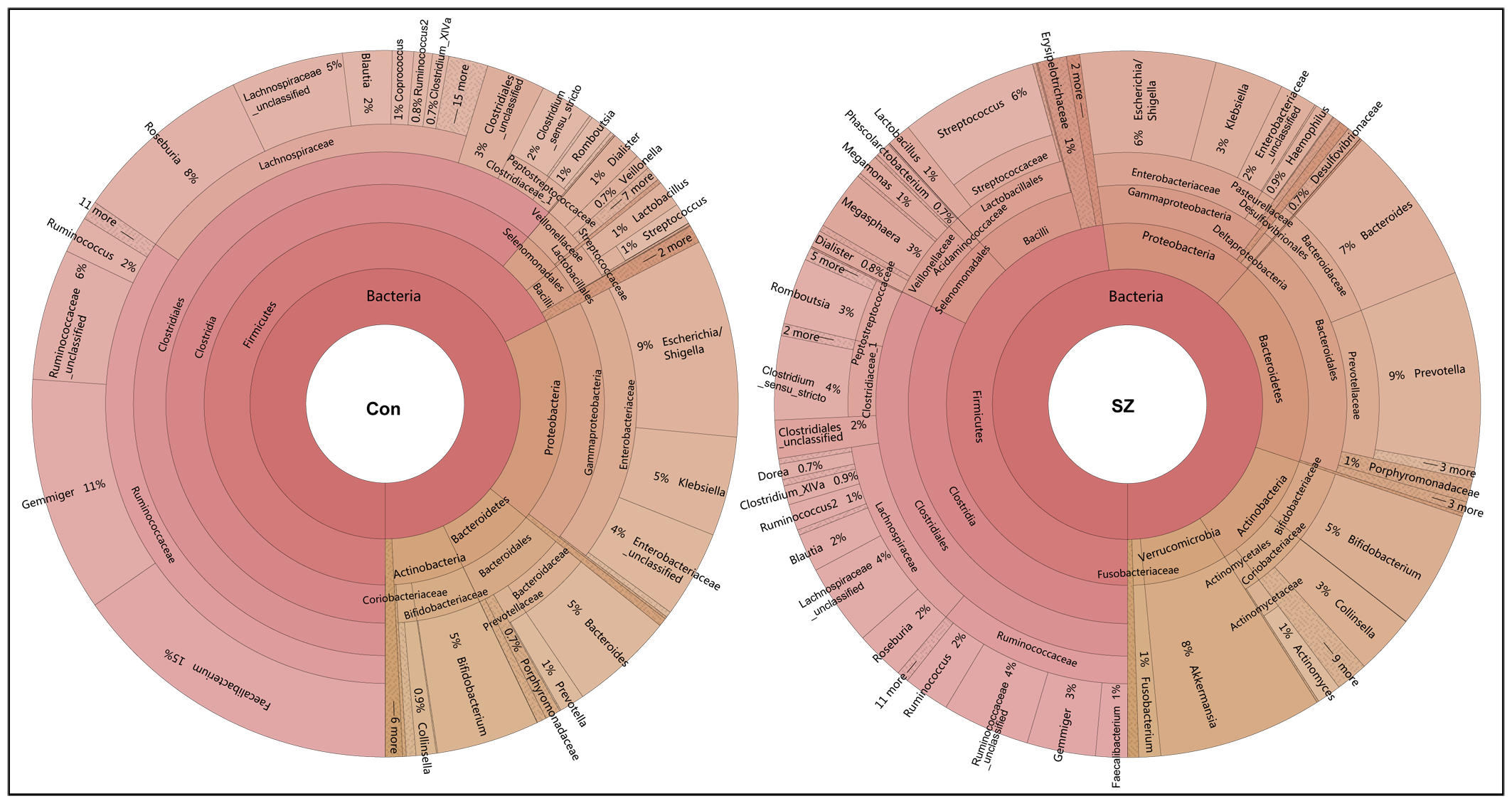

Supplement: Supplementary Figure 2 — Krona charts showing the fecal microbiota in elderly SZ patients and healthy controls. These taxa represent the internal core microbiota at the individual level. [file Image_2.tif]
